# Stability of Commercially Available Macular Carotenoid Supplements in Oil and Powder Formulations

**DOI:** 10.3390/nu9101133

**Published:** 2017-10-17

**Authors:** David Phelan, Alfonso Prado-Cabrero, John M. Nolan

**Affiliations:** Nutrition Research Centre Ireland, School of Health Science, Carriganore House, Waterford Institute of Technology, West Campus, Waterford X91 K236, Ireland; APRADO-CABRERO@wit.ie (A.P.-C.); John@ivr.ie (J.M.N.)

**Keywords:** macular carotenoid supplementation, lutein, zeaxanthin, *meso*-zeaxanthin, macular pigment

## Abstract

We previously identified that the concentration of zeaxanthin in some commercially available carotenoid supplements did not agree with the product’s label claim. The conclusion of this previous work was that more quality assurance was needed to guarantee concordance between actual and declared concentrations of these nutrients i.e., lutein (L) zeaxanthin (Z) and *meso*-zeaxanthin (MZ) in commercially available supplements. Since this publication, we performed further analyses using different commercially available macular carotenoid supplements. Three capsules from one batch of eight products were analysed at two different time points. The results have been alarming. All of the powder filled products (*n* = 3) analysed failed to comply with their label claim (L: 19–74%; Z: 57–73%; MZ: 83–97%); however, the oil filled soft gel products (*n* = 5) met or were above their label claim (L: 98–122%; Z: 117–162%; MZ: 97–319%). We also identified that the carotenoid content of the oil filled capsules were stable over time (e.g., L average percentage change: −1.7%), but the powder filled supplements degraded over time (e.g., L average percentage change: −17.2%). These data are consistent with our previous work, and emphasize the importance of using carotenoid interventions in oil based formulas rather than powder filled formulas.

## 1. Introduction

**Figure 1:**
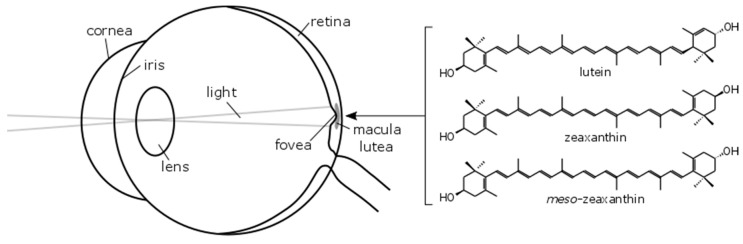
Schematic of the human eye showing the approximate location of the macular pigments and the chemical structure of the carotenoids which comprise macular pigment.

Three macular carotenoids, lutein (L), zeaxanthin (Z) and *meso*-zeaxanthin (MZ) accumulate in the central retina (macula), where they are collectively known as macular pigment (MP) or the macular carotenoids [1] (See Figure 1).

The macula is the area of the retina which has the highest visual performance in terms of visual acuity i.e., motion detection, colour perception and contrast sensitivity [2]. MP has a distinctive yellow colour due to the presence of the macular carotenoids. MP has short-wavelength (blue) light filtering, antioxidant, and anti-inflammatory properties [3,4,5]. The filtration of blue light at the macula is believed to enhance visual performance, due to the attenuation of chromatic aberration; veiling luminescence and blue haze [6,7,8]. Furthermore, MP actively quenches free radicals, and this antioxidant capacity is optimised when all three macular carotenoids are present [9]. L and Z are consumed in a typical diet from sources such as fruits and leafy green vegetables [10,11]. MZ is not present in a typical diet, although it has been detected in shrimp, fish and turtle [12], and also in the liver of frog and quail [13]. More recently MZ has been identified in trout skin and trout flesh [14,15], thereby confirming the presence of this carotenoid in the human food chain [16].

A large number of published studies have tested the efficacy of macular carotenoid supplementation (dietary and food supplementation), on serum and tissue response. The majority of these trials demonstrated a significant response in serum and target tissue (i.e., the retina). This tells us that we are not obtaining optimal amounts of these carotenoids from our standard diet. Hence, and given the known health benefits of these carotenoids for human health (e.g., visual performance and cognitive function) [17], it appears that there is a role for supplementation with these nutrients. For example, we know that supplementation with the macular carotenoids increases MP and enhances visual performance in diseased and non-diseased eyes, and clinical studies have shown that a combination of the macular carotenoids is the best way to achieve this goal [18,19,20,21,22,23,24,25,26]. Importantly, laboratory studies conducted on commercially available macular carotenoid supplements have shown that the capsule contents do not always comply with the product label claim [27]. A previous publication by our group investigated the concordance between declared and actual concentrations of the macular carotenoids in commercially available formulations [15]. In that study, MZ was present in six of seven products which did not declare MZ on their label. Also, concentrations of Z ranged from 47 to 248% of label claim. This has also been shown by Breithaupt et al. (2005), who reported that half (*n* = 14) of the products they tested did not meet label claim [28]. Therefore, the obvious question to address here is why some commercially available food supplements fail to meet their label claim. In the first instance, it appears that the regulation for food supplements is very weak, and attention to quality and stability has been shown to be neglected [15]. Of note, the Food and Drug Administration (FDA) does not require manufacturers of food supplements to include an expiration date on the label of these products [28].

**Figure 2:**
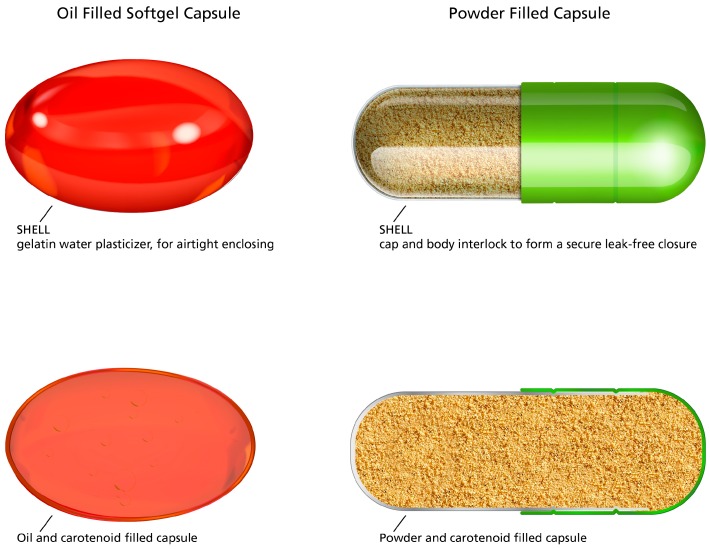
Graphic representation of an oil filled soft gel capsule and a two part powder filled capsule.

Also, there are many factors which are known to affect the stability of carotenoid supplements, including product capsule and product matrix. The capsule type will have an impact on the stability of the product. Soft gel capsules are manufactured using a process where the fill and the shell are manufactured in one operation. It is known that the oil will limit oxidation and enhance the stability and therefore shelf life of the carotenoid [29]. Also, the manufacturing process for oil filled soft gels provides an inert environment during manufacturing (i.e., nitrogen blanketing for oxygen sensitive compounds), and a thorough de-aeration of the fill formulation to remove any dissolved air (oxygen) [30]. This process is extremely important for the preservation of these oxygen sensitive compounds [9,31]. In contrast, carotenoid supplements prepared as powder formulations are subject to oxidation, as there is no oil present to prevent direct contact with oxygen. Also, powder filled capsules are prepared simply by filling one side of the capsule with carotenoid and filler, and then pushing the other side to close the capsule. As these powder filled capsules are not fully sealed, the contents are subject to oxidation from the oxygen within the capsule and potentially from oxygen entering the capsule (See Figure 2).

The aim of this study was to measure the concentration of L, Z and MZ in commercially available food supplements. This work allowed us to compare concordance to label claim for powder and oil filled carotenoid containing capsules. Also, we assessed the stability of the carotenoid contents of these formulations. This work is important, given the vast amount of carotenoid products available, and given that the regulation for carotenoid products is currently not robust. All the supplements analysed in this study contained carotenoid concentrates in their free form.

## 2. Materials and Methods

### 2.1. Supplements Analysed

In this experiment, we analysed two types of commercially available carotenoid supplements, namely oil filled soft gel capsules and powder filled capsules. As we were analyzing commercially available products from a number of manufacturers, it was not possible to align expiration dates to provide a uniform stability study and as such we were limited to analyzing and reanalyzing the products without a uniform time to expiry from date of analysis. The oil filled soft gel capsules analysed included: MacuSave (Zeon healthcare Ltd., Oxfordshire, UK), Doctor’s Best (Doctor’s Best Inc., San Clemente, CA, USA), Lutigold Extra (Puritans Pride Inc., Oakdale, NY, USA), MacuHealth LMZ^3^ (MacuHealth, Birmingham, MI, USA), and MacuShield (Alliance Pharma PLC, Wiltshire, UK).

The powder filled capsules analysed included: MacuSafe (OcuSci, Del Mar, CA, USA), Eye Vitality Plus (Green Valley Hyperion LLC, Lexington, VA, USA) Vision Alive (Holistic Labs Ltd., Hollywood, FL, USA), and MaxiVision (MedOp Health Inc., Oldsmar, FL, USA). The MaxiVision product is a powder filled beadlet formulation. For this formulation, we used a different analytical method, see Section 2.4.

### 2.2. Macular Carotenoid Standards and Solvents

L standard [(3R,3′R,6′R)-β,ε-Carotene-3,3′-diol] and Z standard (racemic mixture of the three Z enantiomers (3R,3′R)-β,β-Carotene-3,3′-diol, (3S,3′S)-β,β-Carotene-3,3′-diol and (3R,3′S)-β,β-Carotene-3,3′-diol) were supplied by CaroteNature GmbH (Ostermundigen, Switzerland). The Standard Reference Material (SRM) 968e (fat-soluble vitamins, carotenoids, and cholesterol in human serum) was obtained from NIST (National Institute for Standards and Technology, Gaithersburg, MD, USA). The solvents THF (tetrahydrofuran), hexane and isopropanol, all HPLC grade, were purchased from Sigma-Aldrich (Vale Road, Arklow, Wicklow, Ireland) or Thermo Fisher Scientific (Blanchardstown Corp Pk 2, Ballycoolin, Dublin, Ireland). BHT (butylated hydroxytoluene) was purchased from Sigma-Aldrich.

### 2.3. Supplement Extraction Method 1: Oil Filled Soft Gel Capsules and Powder Filled Capsules

Sample extraction and preparation were performed under amber light provided by LED lamps installed in our laboratory (Philips BCP473 36xLED-HB/AM 100–277 V) in order to prevent carotenoid isomerization. The antioxidant BHT was added to the extraction solvents to prevent carotenoid degradation. Each supplement was analysed in triplicate i.e., 3 individual capsules were analysed. Single capsules were selected at random and placed in separate 50 mL falcon tubes along with 10 mL of THF. Powder capsules were separated by hand and the contents added to the Falcon tube and an additional 10 mL of THF was added. Oil filled gelatin capsules were pierced with a blade and the contents of the capsule were allowed to mix with the solvent. The blade was washed with 10 mL of THF to reach a final volume of 20 mL. Each tube was vortexed for 10 s, sonicated at 24 °C for 2 min and vortexed again for 10 s, in order to efficiently separate the capsule contents from the shell. The tubes were centrifuged at 4700 rpm at 25 °C without brake to avoid resuspension of the pellet. Dilutions of each tube were then prepared. An aliquot of each dilution was dried in a centrifugal vacuum concentrator (GeneVac MiVac Duo Concentrator, Ipswich, UK) and re-suspended in HPLC mobile phase. The HPLC method is thoroughly described in a previous publication from our group [15]. All products were stored at room temperature in cardboard boxes prior to analysis and between analyses.

### 2.4. Supplement Extraction Method 2: Beadlet Analysis

Sample extraction and preparation were performed under amber light. The Beadlet products capsule and contents were added to a 50 mL Falcon tube, and then 5 mL of 4% (*w*/*v*) ammonium chloride buffer (pH adjusted to 8.6) was added to the capsule contents along with 200 µL of protease K enzyme. Finally 20 mL of THF with 0.1% BHT was added. Each tube was vortexed for 10 s, sonicated for 40 min at 50 °C and then vortexed again for 10 s, in order to efficiently separate the macular carotenoids from the beadlets and other formulation components. The tubes were centrifuged at 4700 rpm at 25 °C without brake to avoid resuspension of the pellet. Dilutions of each tube were then prepared. An aliquot of each dilution was dried in a centrifugal vacuum concentrator and re-suspended in HPLC mobile phase. One capsule only was used for this analysis. This method was adapted from personal communications received from Professor Neal Craft, President of Craft Technologies.

### 2.5. High Performance Liquid Chromatography

The analytical method to analyse supplements has been previously published by our group [15]. L, Z and MZ were separated and quantified on an Agilent Technologies (Palo Alto, CA, USA) 1260 Series HPLC system equipped with a Diode Array Detector (DAD, G1315C), binary pump, degasser, thermostatically controlled column compartment, thermostatically controlled high-performance autosampler (G1367E) and thermostatically controlled analytical fraction collector. For system control and data processing, the software ChemStation (Agilent Technologies) was used. The standard injection volume was 10 µL. Carotenoid quantification was performed using a Daicel Chiralpak IA-3 column, composed of amylose tris (3,5-dimethylphenylcarbamate) bonded to a 3 μm silica gel (250 × 4.6 mm i.d.; Chiral Technologies Europe, Cedex, France). The column was protected with a guard column containing a guard cartridge with the same chemistry of the column. Isocratic elution was performed with hexane and isopropanol (90:10, *v*/*v*) and a flow rate of 0.5 mL min^−1^. The column temperature was set at 25 °C. The LOQ for L and Z was assessed as the lowest concentration which achieved less than 5% RSD from 9 replicate injections (L = 57.4 pmol, Z = 3.3 pmol) [32].

## 3. Results

### 3.1. Oil Filled Soft Gel Capsules

**Figure 3:**
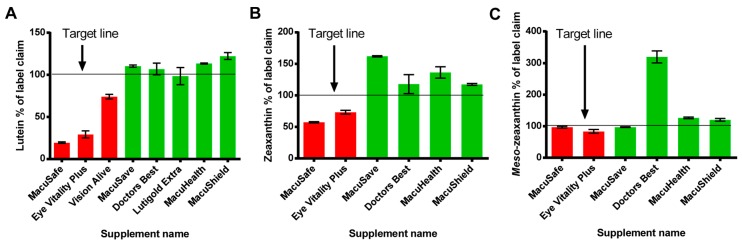
Initial time point analysis illustrating concentration of L (**A**) Z (**B**) and MZ (**C**) expressed as a % of label claim for each product analysed (powder filled capsules are in red, oil filled capsules are in green). Vision Alive and Lutigold products are not shown in (**B**) and (**C**), as these products made no label claim regarding the concentration of Z and MZ. All supplements were analysed in triplicate.

**Figure 4:**
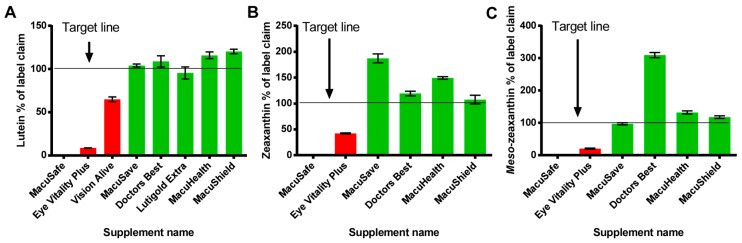
Second time point analysis illustrating concentration of L (**A**) Z (**B**) and MZ (**C**) expressed as a % of label claim for each product analysed (powder filled capsules are in red, oil filled capsules are in green). Vision Alive and Lutigold products are not shown in (**B**) and (**C**) as these products made no label claim regarding the concentration of Z and MZ. All supplements were analysed in triplicate.

All the oil filled soft gel capsules were in close concordance with their label claim for L (98–122%); however, Z and MZ concentration in these supplements were either close to or consistently higher than label claim (Z: 117–162% and MZ: 97–319%). These data are presented in Table 1 and Table 2 as well as Figure 3 and Figure 4. One company, Puritans Pride, who sell the Lutigold Extra supplement, declared Z and MZ on the product label, but did not specify the concentration of these carotenoids.

### 3.2. Powder Filled Capsules

For powder filled capsules, none of the products achieved label claim for L (19–74%), Z (57–73%) or MZ (83–97%). These data are presented in Table 1 and Table 2 as well as Figure 3 and Figure 4. One company, Holistic labs Ltd. (Hollywood, FL, USA) who sell the Vision Alive supplement did not declare Z or MZ on the product label, but these carotenoids were present in their supplement (albeit in small concentrations).

### 3.3. Powder Filled Beadlet Capsules

The carotenoid contents of this product (MaxiVision) did meet or were above their label claim amount for L, Z and MZ (108.2%, 184.0% and 111.9% respectively, see Table 1 and Figure 3). However, in order to extract the carotenoids from this formulation, we needed to use a more aggressive extraction technique (see above, Section 2.4).

### 3.4. Stability of Carotenoid Supplements

To determine if the products were stable over time, we reanalyzed the same batch of each supplement (from the same container) after a number of months (see Table 2). In brief, the oil filled carotenoid supplements yielded similar results to the initial analysis; but the powder filled capsules exhibited a reduction in carotenoid concentration (see Table 2 and Figure 4).

## 4. Discussion

Our experiment confirms that oil filled carotenoid products match or are above their label claim, whereas powder filled carotenoid products do not meet their label claim. Moreover, we identified that oil filled carotenoid products are stable overtime, but powder filled carotenoid products are not.

One of the powder filled carotenoid products (MacuSafe), which the initial analysis showed was not in agreement with the products label claim (% of label claim: L:19.2%, Z:57.0% and MZ: 96.6%) degraded to 0% carotenoid content for each carotenoid within five months, and therefore patients consuming this product would have received no carotenoid intervention/benefit. Of note, this product was still within its expiry date (Expiry June 2019). Also, the other powder filled products degraded over time, but the oil filled soft gel gelatin capsules were stable over time. The results obtained from this experiment are consistent with previous research performed on commercially available carotenoid supplements [15,27,33]. In these previous studies, a large proportion of the supplements analysed did not meet label claim; also, MZ and Z were present in supplements tested although these nutrients were not listed on the label of the product. As observed previously by our group, this experiment identified a large discrepancy between the amount of Z claimed on the product label (for oil and powder). For example, Prado-Cabrero reported that the Z% of label claim varied between 47 and 248%, and in the current study we report that the Z% of label claim ranged from 57 to 319%. The most likely explanation for the lower than claimed concentrations of the carotenoids in these supplements is due to degradation caused by oxidation. It is known that carotenoids react with oxygen via one of three possible mechanisms: oxidation, electron transfer or hydrogen abstraction [34,35]. Interestingly, this is consistent with our results as in the powder formulations L appeared to be consistently lower than MZ and Z in these products, and also appeared to be less stable over time (from our reanalysis, Table 2). In other words, it appears that L is more susceptible to oxidation than Z and MZ and this finding is consistent with the chemistry of the molecules. In brief, the structure of the Z and MZ carotenoid isomers makes them less sensitive to oxygen due to the presence of a double beta ring, whereas in L the presence of a beta and an epsilon ring appears to make this carotenoid more reactive to oxygen [36].

It is likely that the products that exhibited higher concentrations of L, Z and MZ than their label claim was simply due to the fact that these companies added more than required at formulation stage. This is known to occur in the food supplement industry where companies endeavor to ensure label claim is met, or (in some cases) where accurate measurement of the formulation is not performed.

Of note, only one of the powder filled formulations exhibited good results, but this formulation (MaxiVision) provides the carotenoids in a beadlet. For food supplements, beadlets are typically used to protect sensitive molecules (e.g., carotenoids) from degradation (e.g., from oxygen) [37]. There are a variety of functions and purposes of beadlets. For example, beadlets may provide for the separate containment of ingredients within the dietary supplement to improve the stability of the entrapped ingredients. However, very little is known about the bioavailability of these beadlet carotenoid formulations, and clinical trials in human subjects are needed to provide evidence regarding the efficacy of these formulations. Indeed, even for our analysis the analytical procedure required to extract L, Z and MZ from the beadlets was far more aggressive than that required for the oil filled soft gel capsule or the powder filled carotenoid capsules. In brief, extraction of the carotenoids from the beadlets required high temperature control (50 °C), long sonication times, and the use of digestive enzymes (protease), parameters not required to extract the carotenoids from the oil or powder formulations.

## 5. Conclusions

The data presented in this study provides important information concerning carotenoid food supplements. We confirm that a number of commerically available carotenoid food supplements do not achieve their label claim. The evidence from this study is that oil filled soft gel capsules are the best way to provide a stable carotenoid supplement for the consumer. At present, clinicians and consumers are not adequately informed via product labelling, and the food supplement industry does not appear to have sufficient regulations in place to protect the consumer from clinically untested, unstable or degraded products. However, it is also important to point out that there are quality and effective carotenoid products on the market, which have scientific evidence to back up their claims of label and efficacy. In other words, it is our view that clinicians and consumers should select macular carotenoid products which have appropriate scientific evidence confirming product stability and efficacy. The current experiment confirms the importance of using carotenoid interventions in oil based formulas, rather than powder filled formulas.

## Figures and Tables

**Table 1:** Macular carotenoid concentration of the commercially available supplements analysed.

Supplement Name	Type	Batch Number	Expiry	Time to Expiry at Time of Testing (Months)	Macular Carotenoid (mg/Capsule)	
					Declared	Measured $\bar{x}$ ± SD
MacuSafe	1	12603	June 2019	28	L→10	1.92 ± 0.09
					Z→2	1.14 ± 0.02
					MZ→12	9.66 ± 0.28
Eye Vitality Plus	1	13577	August 2019	31	L→15	4.35 ± 0.42
					Z→2	1.46 ± 0.06
					MZ→10	8.32 ± 0.59
Vision Alive	1	424951	June 2018	14	L→10	7.38 ± 0.27
					Z→nc	1.24 ± 0.02
					MZ→nc	1.58 ± 0.04
MacuSave	2	3001777	August 2019	35	L→10	11.01 ± 0.13
					Z→2	3.23 ± 0.02
					MZ→10	9.67 ± 0.11
Doctors Best	2	16052523A	December 2019	32	L→20	21.33 ± 1.39
					Z→2	2.35 ± 0.30
					MZ→1	3.19 ± 0.19
Lutigold Extra	2	462536-02	August 2019	27	L→20	19.65 ± 1.02
					Z→nq	2.14 ± 0.15
					MZ→nq	3.05 ± 0.11
MacuHealth	2	C1600284	September 2019	25	L→10	11.32 ± 0.06
					Z→2	2.72 ± 0.18
					MZ→10	12.60 ± 0.26
MacuShield	2	120480	March 2017	4	L→10	12.24 ± 0.41
					Z→2	11.98 ± 0.43
					MZ→10	13.21 ± 0.48
Maxivision *	3	33690818	None	No expiry date on product	L→10	10.82
					Z→2	3.68
					MZ→10	11.19

1 = powder filled capsule 2 = oil filled soft gel gelatin capsule 3 = powder filled beadlet formulation. nc = not claimed on product label n/a = not applicable nq = not quantified on label * Supplement extraction method 2: Beadlet analysis (*n* = 1). The oil filled soft gel capsules analysed included: MacuSave (Zeon healthcare Ltd., Oxfordshire, UK), Doctor’s Best (Doctor’s Best Inc., San Clemente, CA, USA), Lutigold Extra (Puritans Pride Inc., Oakdale, NY, USA), MacuHealth LMZ^3^ (MacuHealth, Birmingham, MI, USA), and MacuShield (Alliance Pharma PLC, Wiltshire, UK). The powder filled capsules analysed included: MacuSafe (OcuSci, Del Mar, CA, USA), Eye Vitality Plus (Green Valley Hyperion LLC, Lexington, VA, USA) and Vision Alive (Holistic Labs Ltd., Hollywood, FL, USA). The beadlet formulation analysed was: MaxiVision (MedOp Health Inc., Oldsmar, FL, USA). One capsule of MaxiVision was analysed; the rest of the supplements were analysed in triplicate.

**Table 2:** Re-analysis of macular carotenoid concentration of the commercially available supplements analysed after storage.

Supplement Name	Storage Time Months	Type	Batch Number	Expiry	Time to Expiry at Time of Testing (Months)	Macular Carotenoid (mg/Capsule)	
						Declared	Measured $\bar{x}$ ± SD
MacuSafe	5	1	12603	June 2019	23	L→10	0.0 ± 0.0
						Z→2	0.0 ± 0.0
						MZ→12	0.0 ± 0.0
Eye Vitality Plus	6	1	13577	August 2019	25	L→15	0.84 ± 0.02
						Z→2	0.84 ± 0.02
						MZ→10	2.00 ± 0.19
Vision Alive	3	1	424951	June 2018	11	L→10	6.48 ± 0.27
						Z→nd	1.14 ± 0.05
						MZ→nd	1.52 ± 0.01
MacuSave	8	2	3001777	August 2019	25	L→10	10.39 ± 0.18
						Z→2	3.74 ± 0.17
						MZ→10	9.69 ± 0.33
Doctors Best	3	2	16052523A	December 2019	29	L→20	21.74 ± 0.65
						Z→2	2.38 ± 0.09
						MZ→1	3.09 ± 0.08
Lutigold Extra	2	2	462536-02	August 2019	25	L→20	19.05 ± 0.70
						Z→nq	1.99 ± 0.08
						MZ→nq	2.97 ± 0.06
MacuHealth	5	2	C1600284	March 19	20	L→10	11.58 ± 0.39
						Z→2	2.98 ± 0.05
						MZ→10	13.21 ± 0.48
MacuShield	3	2	120480	March 2017	1	L→10	11.75 ± 0.41
						Z→2	2.15 ± 0.17
						MZ→10	12.02 ± 0.27

1 = powder filled capsule 2 = oil filled soft gel gelatin capsule. nd = not detected. Storage time in months is the time between initial analysis and subsequent re-analysis. The oil filled soft gel capsules analysed included: MacuSave (Zeon healthcare Ltd., Oxfordshire, UK), Doctor’s Best (Doctor’s Best Inc., San Clemente, CA, USA), Lutigold Extra (Puritans Pride Inc., Oakdale, NY, USA), MacuHealth LMZ^3^ (MacuHealth, Birmingham, MI, USA), and MacuShield Alliance Pharma PLC (Wiltshire, UK). The powder filled capsules analysed included: MacuSafe (OcuSci, Del Mar, CA, USA), Eye Vitality Plus (Green Valley Hyperion LLC, Lexington, VA, USA) and Vision Alive (Holistic Labs Ltd., Hollywood, FL, USA). All supplements were analysed in triplicate.
